# Comics as an educational tool for children with juvenile idiopathic arthritis

**DOI:** 10.1186/s12969-017-0198-5

**Published:** 2017-09-02

**Authors:** Amir Mendelson, Noa Rabinowicz, Yonit Reis, Gil Amarilyo, Liora Harel, Philip J Hashkes, Yosef Uziel

**Affiliations:** 1 Pediatric Rheumatology Unit, Department of Pediatrics, Meir Medical Center, 59 Tschernihovsky St, 44281 Kfar Saba, Israel; 2 Pediatric Rheumatology Unit, Schneider Children’s Medical Center, Petach Tikvah, Israel; 3 Sackler School of Medicine, Tel Aviv University, Tel Aviv, Israel; 4 Pediatric Rheumatology Unit, Shaare Zedek Medical Center, Jerusalem, Israel; 5 Hebrew University Medical School, Jerusalem, Israel

**Keywords:** Education, Knowledge, Juvenile idiopathic arthritis, Comics, Patient education

## Abstract

**Background:**

This study examined whether the comic book *Neta and the Medikidz Explain JIA* would improve disease-related knowledge and treatment adherence among patients with juvenile idiopathic arthritis (JIA).

**Methods:**

In this prospective cohort study, JIA patients answered 20 multiple-choice knowledge questions about their disease, before and after reading the comic book. Demographic, clinical, health-related quality of life and adherence data were recorded and correlated to the responses.

**Results:**

We studied 61 patients with a mean age of 14 ± 3.3 (range 8–18) years, 67% female, 83% Jewish and 17% non-Jewish. Thirty-nine percent had oligoarthritis, 13% systemic, 32% polyarthritis 11% psoriatic and 5% enthesitis-related type JIA. The disease was active in 46%, 40% were treated with biologics/disease modifying anti-rheumatic drugs, and 34% were in remission on medication. Among the 53 patients who completed before and after quizzes, average score increased from 63 to 80% (*P* < 0.001). Non-Jewish patients initially scored lower than Jewish patients (48%), but their score increased to 79% after reading the comic book. Twenty-seven patients who also completed the quiz 1 year after the first reading retained their knowledge (79%). We did not find a statistically significant correlation between knowledge and age, sex, disease subtype, or Child Health Questionnaire quality of life scores. Adherence to medication use, physical therapy and rheumatology clinic visits were high at baseline; thus, these did not change after reading the comic.

**Conclusions:**

The comic booklet *Neta and the Medikidz Explain JIA* is a good educational tool for increasing disease-related knowledge in children with JIA.

## Introduction

Juvenile idiopathic arthritis (JIA) is the leading chronic rheumatic disease of childhood and may cause short and long-term morbidity [1, 2]. Adherence to the comprehensive treatment plan is crucial to achieving disease control.

Several factors can affect adherence to treatment, including developmental aspects such as age, cognitive and emotional elements, fear, guilt, low self-esteem and family-related factors. Medical issues such as type and severity of the disease, treatment regimen, and interactions between the patient and the physician are also important [3]. Disease knowledge is another major component of adherence. Studies in children and adolescent patients, especially for diabetes, show that increased knowledge improves adherence and health status [4–6].

The field of Medical Education is continuously changing to accommodate new types of media. Medical knowledge can be delivered through many modes, including the Internet and social networks [7, 8].

Comics were established as a literary medium by the 18th century [9]. In addition to entertainment, comics promote discussion in many subject areas, including philosophy and political revolutions [9]. They have also been used in medicine to demonstrate disease narratives and to serve as a tool for learning about diseases, feelings, emotions, and environmental experiences [10]. The combination of text and figures may increase the effectiveness of education by activating different data management pathways in the brain [11].

Medikidz, a medical education organization for children, has developed comic books to support children’s education with regards to their health and well-being [12]. Topics include eczema, asthma, kidney disorders, tumors, and explanations regarding tests and participation in clinical studies. The comic book *Neta and the Medikidz explain JIA* has been published internationally in several languages, including Hebrew.

Our hypothesis was that reading the book would increase knowledge and improve adherence to the treatment plan. Hence, our aims were to investigate whether the comic book would improve knowledge regarding JIA in the short-term, with long-term retention, and to assess whether increased knowledge would improve adherence with the treatment plan, and improve the participants’ physical and psychological health-related quality of life.

## Methods

Children with JIA, 8 to 18 years of age, who are followed in the pediatric rheumatology clinics in 3 medical centers in Israel (Meir Medical Center, Schneider Medical Center and Shaare Zedek Medical Center), participated in this prospective, cohort study. They answered 20 multiple choice knowledge questions (Appendix A) about JIA before, approximately 1 month and 1 year after reading the comic book *Neta and the Medikidz explain JIA*. The first “pre-reading” questionnaire was given in the clinic. Most answered it in the clinic and some answered it at home and mailed it back together with the second “within 1 month after reading” questionnaire. The third “1-year follow-up” questionnaire was given in the clinic; some answered it in the clinic but most took it home and mailed it back. Parents were instructed not to help with the answers. They were allowed to assist by translating or explaining the questions. Possible scores ranged from 0 to 20, which were scaled from 0 to 100%. Data regarding demographics, disease type and disease activity level were abstracted from electronic medical records. Health-related quality of life data was obtained from the Childhood Health Questionnaire Parent Form 50 (CHQ-PF50).

Treatment adherence was assessed by combining 3 parameters. A scale of 1 (low) to 5 (high) was used to analyze each of the 3 parameters indicating adherence: (1) medication use (we asked the parents whether they took the medications and in parallel checked the pharmacy medication disbursement records), (2) attending scheduled physiotherapy appointments and (3) arriving at scheduled clinic visits. We did not analyze adherence based on the type of medication or side effects, as adherence was high initially.

The Institutional Review Board in 2 of the 3 hospitals exempted the study from approval, as this was a questionnaire-based study only. In the third hospital, the study was registered and parents completed the informed consent forms.

### Statistical analysis

Continuous parameters are presented as means and standard deviations, and nominal parameters are presented as numbers and percentages. Nominal parameters were evaluated with chi-square test. Data from normally distributed continuous parameters were analyzed using paired T-test and the Wilcoxon non-parametric test was used for data that did not have a normal distribution. Correlations between continuous parameters were evaluated by Pearson or Spearman Correlation, each according to the data distribution. Differences between groups were assessed by T-test or Mann-Whitney, as above. A statistical difference was defined when *p* < 0.05. All statistical analyses were conducted using SPSS-22.

## Results

A total of 61children with JIA answered the quiz before reading the comic book. Their mean age was 14 ± 3.3 (range 8–18) years and 67% were female. Most were Jewish (83%). The average disease duration was 64.8 ± 50.4 months (median 55.2 months and range 4 to 177). Among the patients’ parents, 62% of the fathers and 64% of the mothers had at least a high school education. Among the participants, 79% answered the quiz by themselves and 21% received help from their parents; 70% read the comic once and 26% had read the comic book 2 to 5 times prior to answering the quiz.

Fifty-three (87%) of the 61 participants also completed the quiz 1 month after reading the comic book, and 27 (44%) completed it again after 1 year. Eight of the one-month follow-up quizzes were lost in the mail. No statistical differences were found between the demographic and disease state data of those who answered the two post-quizzes and those that did not.

Among the participants 39% had oligoarthritis, 13% systemic arthritis, 32% polyarthritis, 11% psoriatic and 5% enthesitis-related-type JIA. Active disease was present in 46% of participants, 34% were in remission with medication, 20% were in remission with no medication, 40% were treated with biologic medications or disease modifying anti-rheumatic drugs (DMARD), 10% were treated with non-steroidal anti-inflammatory drugs (NSAID) only and 12% were treated with NSAIDs and intra-articular corticosteroid joint injections.

Table 1 describes the baseline knowledge and changes after reading the comic book. The mean correct score rate prior to reading the comic among 61 children was 63% ± 7%. Among the 53 who answered the quiz within 1 month after reading the booklet, correct responses increased to 80% ± 16% (*P* < 0.0001).

**Table 1:** Knowledge quiz results before and after reading the comic book

Time frame	N	Mean score^a^	*P*-value
Before	61	63%	
After 1 month	53	80%	<0.0001
After 1 year	27	79%	

*SD* standard deviation; Min, minimum, *Max* maximum

^a^ Scores ranged from 0 to 20, scaled from 0 to 100%

At each time point, no correlation was found between knowledge differences and participant sex, age (below <14 or ≥14 years old), parental education (high school or above), CHQ-PF50 score or disease subtype. Prior to reading the comic book, Jewish children had significantly higher knowledge scores than non-Jewish children (64% vs. 48%) respectively; *p* = 0.018). Knowledge improved significantly after reading the comic book regardless of JIA status. Among the 27 children who answered the quiz again after 1 year, the mean correct answer score was 79% ± 20%, indicating they had retained the knowledge.

Table 2 describes treatment adherence before and after reading the comic. Adherence was high prior to reading the comic and remained high at one-year follow-up. Adherence with physical therapy decreased slightly in conjunction with improvement in disease activity.

**Table 2:** Adherence with medication, physical therapy and clinic visits according to time from reading the comics

Time frame	Adherence^a^		
	Medication	Physical therapy	Clinic visits
Before	4.5 ± 1.1	4.6 ± 0.8	± 4.7 0.6
3 months after	4.6 ± 1.2	4.1 ± 1.2	± 4.8 0.4
6 months after	4.6 ± 0.9	3.6 ± 1.7	0.5 ± 4.8
1 year after	4.7 ± 0.9	3.6 ± 1.4	1 ± 4.6

^a^ Scale of 0–5 (5 is highest adherence)

## Discussion

The results of this study indicate that knowledge regarding JIA increased significantly after reading the comic book *Neta and the Medikidz explain JIA* among children who have this disease. The mean score improved from 63 to 80% (*P* < 0.001) and the information was retained after 1 year. The increase in knowledge was even more impressive among non-Jewish patients, who demonstrated less knowledge at baseline. Most of these patients do not speak Hebrew as their first language, as opposed to their physician. Pediatric patients who speak Arabic and not Hebrew communicate less with the doctors, and are aided by their relatives (parents or other) to overcome this language barrier. It appears that this population benefitted most from the comic. As it is a tool that combines illustrations and text, it taught them about their disease in a way that regular visits with the doctor (who does not speak Arabic) did not. Comics have been an entertainment and educational medium since the early twentieth century [9]. As an educational tool, it was shown to improve children’s knowledge in several diseases. This was the first study to examine this effect in JIA.

Our results agree with those of similar studies that evaluated comics as an educational medium. In a Japanese study, 219 participants, ages 10–11 years old [13], and in another study, children ages 13–15 years old [14], listened to a lecture about cerebro-vascular stroke that included comics and animation. Participants were better able to identify a stroke and had a higher level of knowledge in this subject. The knowledge was retained when tested 3 months later [13, 14].

A study conducted in parallel in the United States and India showed that children 5–7 years of age who learned about the dangerous effects of fire through comics improved their knowledge significantly [15]. In another study conducted in Spain among 231 eight-year-old children, the effect of comics as an educational tool for preventing lower back pain was assessed. A slight increase in knowledge was obtained, which was retained 3 months after the reading the comic book [16]. A recent, randomized, controlled, parallel-group study demonstrated that reading a comic book about pediatric anesthesia before surgery resulted in significantly lower preoperative anxiety [17].

Medical knowledge regarding a disease is acquired during interactions between patient and physician and the paramedical staff. Printed material about different diseases, written by various sources is often available in the clinic. Abundant information can be found on the Internet, as well. The PRINTO web site [8] is a very detailed, evidence-based source for pediatric rheumatology. However, this information is intended mainly for parents/caretakers, and occasionally for older adolescents. Educational tools for young children with autoimmune diseases are lacking; thus, they must rely on local or national initiatives. Comic books, such as those published by *Medikidz* (http://www.medikidz.com/) for a variety of diseases, help fill this need. We encourage PRINTO to add material in various languages suitable for children, accompanied by illustrations that can teach them about their disease, as in the comic book.

In addition to the increase in disease knowledge demonstrated in the above studies, it seems that treatment adherence improves after reading educational comics. For example, increased adherence was demonstrated in young adolescents with diabetes after reading the comics [4, 5].

We evaluated adherence by measuring 3 parameters, in scales of 1 (lowest) to 5 (highest): medication use, attending physiotherapy visits and attending clinic visits. Due to a high baseline adherence rate, our study did not show a significant increase in adherence. The few patients with lower baseline adherence improved their adherence, but due to their small numbers this did not reach statistical significance.

A limitation of the study was the method of collecting the quiz data. Some follow-up quizzes which were given in the clinic and not filled at the visit, were unfortunately not returned by mail, so the number available for analysis was lower than it was at the beginning of the study. In addition, we cannot rule out that patients might have looked up answers for the 2 post-reading questionnaires (the 1 month and the 1-year questionnaires were both completed at home, in most cases). As the results varied (not everyone scored 100%), we believe this indicates that this was unlikely.

## Conclusions

The comic book *Neta and the Medikidz explain JIA* led to increased knowledge about the disease. The assumption that formed the basis of this study, that a patient who knows more about his/her disease will increase adherence was not proven, as the initial adherence among out patients was high. Therefore, we would like to evaluate the use of comic books or other educational tool in a larger population, with poorer initial medication compliance.

## Appendix

### Quiz on Juvenile Idiopathic Arthritis (JIA)

1. What is an autoimmune disease?

A. A disease of automobiles.
B. A disease developed by children who are active in sports.
C. A disease in which the immune system is confused and attacks our body.
D. A flu-like disease.
E. A disease caused by low functioning of the immune system.

2. How can you get infected with juvenile idiopathic arthritis (JIA)?

A. By drinking from a cup of a sick person.
B. By touching a sick person’s wound.
C. By giving “high five” to a sick person.
D. You cannot get infected or infect another person.

3. Which parts of the joint are affected in juvenile idiopathic arthritis (JIA)?

A. The bone.
B. The cartilage and inner layer of the capsule.
C. The synovial (joint) fluid and the outer layer of the capsule.
D. The white blood cells (leukocytes).

4. Which statement is ***not*** true about the function of the synovial (joint) fluid and the cartilage?

A. They are part of the blood flow.
B. They are present in the capsule and maintain smooth movement of the joints.
C. They enable the bones to slide on each other without bumping into each other.
D. They pad the ends of bones.

5. What happens to the cartilage in juvenile idiopathic arthritis (JIA)?

A. The cartilage wears out and becomes thin.
B. The cartilage becomes hard.
C. The cartilage fights the cells of the immune system.
D. The cartilage gets swollen.

6. What causes the joints to become swollen and hard?

A. The accumulation of stones inside the joints.
B. Collection of air inside the synovial (joint) membrane.
C. Collection of immune cells, thickening of the capsule and increased synovial fluid.
D. Blood collected at the joints.

7. What causes JIA?

A. The immune cells attack the inner parts of the joints.
B. A bacteria or a virus attacks the joints.
C. Usually the joint gets injured and this starts JIA.
D. The “JIA bacteria” attack the white cells (leukocytes).

8. What is true about the joints in our body?

A. They wrap around the muscle.
B. They are the meeting point of bones and let us move and bend.
C. You can’t see them on an x-ray.
D. They include the knees, elbows, eyes and teeth.

9. Why does the immune system attack the joints?

A. During the winter the blood cells and joints shrink and are exposed to damage.
B. Because the body is weak after sports class.
C. Because during the night the body is sleeping and the immune army system is sleeping.
D. It is not known, which is why it’s called idiopathic – unknown reason.

10. Symptoms of JIA could be?

A. Stiff and swollen joints.
B. High fever.
C. Skin rash.
D. All the above.

11. Which tests can be done to diagnose JIA?

A. Blood and urine test.
B. Physical exam, height, blood pressure and weight.
C. Physical exam, blood test, x ray, MRI and ultrasound.
D. Muscle biopsy and x ray.

12. What is true about the different sub-types of JIA?

A. Oligoarticular – affects 4 joints or less, Polyarticular – affects many small and big joints, Systemic- affects many joints and in addition there is fever and rash.
B. Oligoarticular – affects the knees only, polyarticular – affects fingers only, Systemic – affect the cervical lymph nodes for a whole month.
C. There are two types: In the oligoarticular type (affects few joints) there is fever, and in the polyarticular type (affects many joints), it is hard to climb stairs.
D. The lightest type is systemic JIA; the most common is many joints (polyarticular).

13. How do the different medications against JIA work?

A. They prevent the immune system cells from attacking the joints, decrease joint swelling and make the immune system calmer.
B. They grease the joints and relax the pain.
C. They fight the bacteria and viruses that are inside the joints.
D. All the above.

14. Which medications treat JIA?

A. Neutrophil, Leukocyte and Dentrite.
B. Acetaminophen, Fenistil and Azythromycin.
C. Non-steroidal anti-inflammatory drugs (NSAIDS), second line drugs that counteract rheumatic diseases (DMARDs), corticosteroids and biologic drugs.
D. Antibiotics, steroids and anti-clotting drugs.

15. Which drugs are given by injections into the joint?

A. Corticosteroids
B. Non-steroidal anti-inflammatory drugs.
C. Biological drugs.
D. Second-line drugs like methotrexate.

16. What side effects can NSAIDS have?

A. Rash and fever.
B. Sore throat.
C. Joint pain.
D. Abdominal pain and nausea.

17. What should you do to be healthy?

A. Go to the nurse and get a vaccine against the disease.
B. Eat pizza, read comic books and watch TV.
C. Do physical exercise and stretches and take the drugs on time.
D. Drink tea and take paracetamol.

18. What is true about physical exercise?

A. Those who have JIA can’t play basketball.
B. The joints need exercise so it is very important to exercise, for example: swimming.
C. When you jump rope, the immune system cells get dizzy and explode.
D. When you stretch the knee, the swelling disappears.

19. Which doctor follows your disease?

A. Dermatologist
B. Rheumatologist.
C. Gastroenterologist.
D. Urologist.

20. Those that get treated for JIA, usually

A. Get worst as they get older.
B. Stay the same.
C. Get a lot better with time.
D. All the children get healthy always.

## Abbreviations

JIA: Juvenile idiopathic arthritis
